# Supplementary material for: Content-rich biological network constructed by mining PubMed abstracts
Source: BMC Bioinformatics. 2004 Oct 8;5:147. doi: 10.1186/1471-2105-5-147 (PMC528731; doi:10.1186/1471-2105-5-147)
Supplement: Additional File 2 — The original results of the above study (non-essential files are deleted to keep the file size under the limit set by BMC bioinformatics). [file 1471-2105-5-147-S2.bz2 › chilibotAdditionalFile2/dip40/265ID7997270E1066/html/MAPK1_ARAF1.html]

 


 **MAPK1** and **ARAF1** 
  
Found 67 abstracts in PubMed, retrieved 40.  
 

 What does Google say? 
 PDF only 
| .edu only 

---

**Interactive relationship** (e.g. stimulation, inhibition, etc)

**Inhibitory relationship**- However, dominant negative A Raf  [ **ARAF1** ]  effectively blocked MEK activation, suggesting that activation of the MEK Erk  [ **MAPK1** ]  signaling cascade is mediated through A Raf  [ **ARAF1** ] .  Ref: 10066754 J Biol Chem, 1999
- the MEK >ERK  [ **MAPK1** ]  pathway inhibits ANF gene expression while a Raf  [ **ARAF1** ]  induced, MEK independent pathway activates expression.  Ref: 10664445 FEBS Lett, 2000
**Stimulatory relationship**- Moreover, Galpha o Q205L failed to affect MAPK  [ **MAPK1** ]  activation by fibroblast growth factor receptors, which stimulate Raf 1 and A Raf  [ **ARAF1** ]  but not B Raf activity.  Ref: 10749919 Mol Biol Cell, 2000
- PI3 kinase inhibitors enhanced CSF 1 stimulated A Raf  [ **ARAF1** ]  and c Raf 1 activities, and dominant negative A Raf  [ **ARAF1** ]  but not dominant negative c Raf 1 reduced CSF 1 provoked ERK  [ **MAPK1** ]  activation, suggesting that A Raf  [ **ARAF1** ]  mediates a part of the stimulatory signal from Ras to MEK ERK  [ **MAPK1** ] , acting in parallel to PI3 kinase.  Ref: 10958675 Mol Cell Biol, 2000
**Neutral relationship**- In these cells, ERK  [ **MAPK1** ]  are activated by a Raf  [ **ARAF1** ]  MEK dependent pathway, a P13K Raf independent pathway and a third undetermined pathway.  Ref: 11146560 Oncogene, 2000
- Raf comprises a family of three kinases, A Raf  [ **ARAF1** ] , B Raf and Raf 1, which are best known as key regulators of the MEK MAPK  [ **MAPK1** ]  ERK  [ **MAPK1** ]  cascade.  Ref: 11950876 J Cell Sci, 2002

**Non-interactive relationship** (e.g. studied together, co-existance, homology, etc.)

- GLP1 stimulated activation of Erk  [ **MAPK1** ]  is blocked by inhibitors of MEK, but GLP1 does not induce the activation of A Raf  [ **ARAF1** ] , B Raf, C Raf, or Ras.  Ref: 12364324 J Biol Chem, 2002
- ERK  [ **MAPK1** ]  signalling and oncogene transformation are not impaired in cells lacking A Raf  [ **ARAF1** ] .  Ref: 11821947 Oncogene, 2002
- CONCLUSION Our findings link, for the first time, the stimulation of a nicotinic acetylcholine receptor by a cancer causing agent with the activation of a Raf 1  [ **ARAF1** ]  MAPK  [ **MAPK1** ]  c myc signaling pathway.  Ref: 11768610 J Cancer Res Clin Oncol, 2001
- To investigate the importance of Raf MEK ERK  [ **MAPK1** ]  signaling in human hematopoietic cell growth, the effects of three different Raf genes, A Raf  [ **ARAF1** ] , B Raf and Raf 1, on cell cycle progression and regulatory gene expression were examined in TF 1 cells transformed to grow in response to beta estradiol regulated DeltaRaf ER genes.  Ref: 12429936 Cell Cycle< MedlineTA>Cell Cycle, 2001
- These studies suggest that the ability of Ras and BCR ABL to activate NF kB involves an uncharacterized pathway which does not involve classic IKK activity and which suppresses the TNF induced IKK pathway through a Raf  [ **ARAF1** ]  MEK Erk  [ **MAPK1** ]  independent mechanism.  Ref: 12851413 J Biol Chem< MedlineTA>J Biol Chem, 2001
- This implies either that A Raf  [ **ARAF1** ]  plays no role in MEK ERK  [ **MAPK1** ]  activation, that its function is fully compensated by other Raf proteins or MEK kinases or that its role in MEK ERK  [ **MAPK1** ]  activation is highly tissue specific.  Ref: 11821947 Oncogene, 2002
- This effect of Src is mediated partly through a Raf  [ **ARAF1** ]  mitogen activated ERK  [ **MAPK1** ]  kinase extracellular signal regulated kinase signaling cascade and partly through a MEKK JNKK JNK cascade.  Ref: 11145737 Mol Endocrinol, 2001
- H. pylori regulates the activity of this element via a Raf 1  [ **ARAF1** ]  MEK ERK  [ **MAPK1** ]  pathway, which was activated in a Ras independent manner.  Ref: 10652359 J Biol Chem, 2000
- We demonstrated previously that in bovine tracheal myocytes, pretreatment with either forskolin or histamine significantly reduces both platelet derived growth factor PDGF and epidermal growth factor induced Raf 1 activation but fails to inhibit extracellular signal regulated kinase ERK  [ **MAPK1** ]  activation substantially, evidence of a Raf 1  [ **ARAF1** ]  independent ERK  [ **MAPK1** ]  activation pathway.  Ref: 10226075 Am J Respir Cell Mol Biol, 1999
- Multiple components of the extracellular signal regulated protein kinase ERK  [ **MAPK1** ]  cascade, including A Raf  [ **ARAF1** ] , c Raf 1, mitogen activated protein kinase kinase, and ERK  [ **MAPK1** ] , were detected in caveolae under resting conditions.  Ref: 10325235 Circ Res, 1999
- ERK  [ **MAPK1** ]  activation through both TPalpha and TPbeta was dependent on PKA and phosphoinositide 3 kinase PI3K class 1 A, but not class 1 B, and was modulated by Harvey Ras, A Raf  [ **ARAF1** ] , c Raf, and Rap1B B Raf and also involved transactivation of the epidermal growth factor receptor.  Ref: 11901221 Mol Pharmacol, 2002
- To investigate the specific role of A Raf  [ **ARAF1** ]  in this process we generated A Raf  [ **ARAF1** ]  deficient mouse embryonic fibroblasts MEFs and embryonic stem ES cells by gene targeting and characterized their ability to undergo proliferation, differentiation, apoptosis, ERK  [ **MAPK1** ]  activation, and transformation by oncogenic Ras and Src.  Ref: 11821947 Oncogene, 2002
- Thus, the suppression of integrin activation is a Raf  [ **ARAF1** ]  kinase induced regulatory event that can be mediated independently of bulk activation of the ERK  [ **MAPK1** ]  MAP kinase pathway.  Ref: 12134066 Mol Biol Cell, 2002
- Selectivity was demonstrated by the lack of effect of ISIS 5132 on A Raf  [ **ARAF1** ]  or ERK  [ **MAPK1** ] , while a random ODN produced only minor effects on growth and did not influence c Raf expression.  Ref: 11742498 Br J Cancer, 2001
- More recently, we have demonstrated that PGF2alpha activates extracellular signal regulated kinase ERK  [ **MAPK1** ]  mitogen activated protein MAP kinase signaling through a Raf  [ **ARAF1** ]  dependent mechanism in bovine luteal cells.  Ref: 11159862 Endocrinology, 2001
- Gastrin activated transcription through a Raf  [ **ARAF1** ] , Mek and Erk  [ **MAPK1** ]  dependent but Ras independent pathway.  Ref: 12891554 Gastroenterology, 2003
